# The effect of different interfaces during virtual game practice on motor performance of individuals with genetic ataxia: A cross-sectional study

**DOI:** 10.1371/journal.pone.0312705

**Published:** 2024-11-01

**Authors:** Zodja Graciani, Íbis Ariana Peña de Moraes, Camila Aparecida de Oliveira Alberissi, Janina Manzieri Prado-Rico, Talita Dias da Silva, Juliana Perez Martinez, Luciano Vieira de Araújo, Rodrigo Garcia Pontes, Susi Mary de Souza Fernandes, Renata Cléia Claudino Barbosa, Andrea H. Németh, Helen Dawes, Carlos Bandeira de Mello Monteiro

**Affiliations:** 1 Postgraduate Program in Rehabilitation Sciences, Faculty of Medicine, University of São Paulo, São Paulo, SP, Brazil; 2 Physical Therapy Department, Mackenzie Presbyterian University, São Paulo, SP, Brazil; 3 Physical Therapy Department, University São Camilo Center, São Paulo, SP, Brazil; 4 NIHR Exeter Biomedical Research Centre, College of Medicine and Health, St Lukes Campus, University of Exeter, Exeter, United Kingdom; 5 Department of Physiotherapy, Federal University of Juiz de Fora Campus Governador Valadares, Governador Valadares, MG, Brazil; 6 Postgraduate Program in Physical Activity Sciences, School of Arts, Science and Humanities, University of São Paulo, São Paulo, SP, Brazil; 7 Department of Neurology, The Pennsylvania State University College of Medicine and Milton S. Hershey Medical Center, Hershey, Pennsylvania, United States of America; 8 Postgraduate Program in Medicine (Cardiology), Federal University of São Paulo, São Paulo, SP, Brazil; 9 Nuffield Department of Clinical Neurosciences, University of Oxford, Oxford, United Kingdom; Isfahan University of Medical Sciences, ISLAMIC REPUBLIC OF IRAN

## Abstract

**Purpose:**

Reaching and coordination tasks are widely used in traditional physical rehabilitation programs for individuals with Ataxia. Virtual reality interventions could optimize the motor performance of these individuals; however, the type of virtual interface may influence performance during virtual practice. We aimed to estimate the extent of the effect of different interfaces (webcam and touchscreen) on the motor performance of individuals with various types of genetic ataxia, compared to a control group, during virtual computer game tasks.

**Methods:**

Repeated exposure quasi-experimental design, which included seventeen volunteers diagnosed with progressive ataxia between 21 and 64 years of age and sixteen age-matched controls. The virtual game tasks were based on the MoveHero software, performed using different interfaces (webcam or touchscreen). Subgroups of participants with genetic ataxia performed the virtual games using the interfaces in different orders (webcam interface followed by touchscreen interface, or vice-versa). The absolute error (AE), variable error (VE), number of hits, and anticipation were used to reflect the motor performance during the virtual task.

**Results:**

Participants with ataxia presented more variable and absolute errors, a lower number of hits, and greater anticipation error than controls (p<0.05). For participants with ataxia, a greater AE was found only in the sequence touchscreen followed by webcam interface (i.e., the sequence webcam before touchscreen presented lower AE).

**Conclusion:**

The group of participants with genetic ataxia presented lower performance than the control group regardless of the interface (webcam or touchscreen). The most interesting observation was that although practicing with the webcam interface offers features that make the task more complex than the touchscreen interface, resulting in lower performance, this interface facilitated performance in a subsequent touchscreen task only in individuals with ataxia, suggesting that a virtual interface engenders greater transfer to other tasks.

Registered at Registro Brasileiro de Ensaios Clínicos (ReBEC) database number identifier: RBR-3q685r5.

## Introduction

Genetic ataxias (GA) are rare conditions, with a world prevalence of 2.7–3.3/100,000 [1, 2]. They are often degenerative diseases with a hereditary basis, and with a prognosis and natural history that depends on the genetic basis, including the specific genetic mutation present and any respective systemic repercussions [3, 4]. Common sensorimotor cardinal manifestations involve, to varying degrees, changes in the speed, amplitude, and synergism of movements associated with dysarthria, dysphagia, dysmetria, nystagmus, tremor, decreased muscle tone, and gait instability [5–8].

The functional repercussions of GA can vary according to the specific condition, but in general, patients present with poor balance, postural instability and lack of motor coordination, affecting gait and gross motor movements [6, 9, 10]. In addition to progressive deterioration of the lower limb function, there is also evidence of more subtle losses in upper limb coordination but with slower progression [11]. A high level of functional dependence with evident progression is also observed in these patients due to the lack of manual dexterity [8].

In order to minimize the effects of motor difficulty in the upper limbs, it is important to develop new rehabilitation approaches. Virtual reality technology is a complement to enhance traditional rehabilitation programs [6, 12, 13]. This technology could quantify and monitor motor dysfunction and simultaneously provide and progress tailored rehabilitation, by allowing for daily fluctuations in ability [14–16].

Some studies support the idea that rehabilitation programs that include virtual reality (VR), can provide gamified enjoyable interventions, lead to better results compared to conventional treatment, through more tailored, and rewarding, exercise prescription [17]. VR platforms can offer safe and reproducible contexts and can simulate activities of daily living [18, 19].

Considering the potential benefits of VR on motor performance and the lack of scientific evidence for using VR in individuals with a degenerative genetic disease, it is of great relevance to conduct studies with virtual interfaces for individuals with balance and motor coordination disorders, such as GA.

Hence, an important gap in clinical practice and research using VR is the influence of the type of interface on learning, improvement in performance, and engagement [20, 21]. Therefore, in the current study, individuals with GA and a control group (organized with able-bodied individuals) practiced a novel VR task to explore the effects of different interfaces on motor performance.

Thus, we aimed to verify whether individuals with GA present differences in motor performance when executing the same task with distinct interfaces (i.e., with physical contact, characterized as touchscreen, or without physical contact, characterized as webcam). Additionally, we identify whether and how practicing a task in a non-contact interface (webcam) influences the following task performance in a contact interface (touchscreen) performed immediately afterward. We hypothesized that: (1) the control group would present better performance in all protocols; (2) a physical contact task (touchscreen) that offered a more efficient processing channel due to tactile feedback would lead to better performance in both groups; and (3) practice in a more difficult environment, characterized by a non-contact interface (webcam) would provide better transfer to a subsequent task with physical contact (touchscreen).

## Methods

This study was approved by the research ethics committee of the University of São Paulo (CAAE: 05221118.9.0000.0065) and was registered in the Brazilian Registry of Clinical Trials (ReBEC; number RBR-3q685r5). Informed written consent was obtained from all subjects involved in the study and the collections were performed after the research participant signed the Informed Consent Form. This cross-sectional study was carried out at the Center of Health Promotion and Rehabilitation and Social Integration of Sao Camilo University Center in São Paulo, São Paulo, Brazil. The study follows the guidelines and recommendations of the Strengthening the Reporting of Observational Studies in Epidemiology (STROBE) [22]. The recruitment of participants was carried out over the 6 months preceding the beginning of data collection, that occurred between December 5, 2022 and June 9, 2023.

### Inclusivity in global research

Additional information regarding the ethical, cultural, and scientific considerations specific to inclusivity in global research is included in the (S1 Checklist).

### Study design

The participants with Ataxia and the able-bodied Control group were randomized into two subgroups, allocated according to the sequence in which they started the protocol, i.e., subgroup T-W started the protocol by performing the virtual task through the touchscreen interface, followed by the same task in the webcam interface. While subgroup W-T performed the opposite sequence: started the protocol with the virtual task through the webcam, followed by the same task in the touchscreen interface.

Block randomization and stratification were chosen to obey homogenization by age, sex, and severity of health condition (the latter in the case of the experimental group), characterizing a repeated exposure quasi experimental design.

### Participants

The study included 33 volunteers, of whom 17 have a clinical and molecular diagnosis of various types of genetic ataxia, without intellectual disabilities. The participants were divided into groups balanced for age (between 21 and 64 years) and clinical profile (functional, visual, and cognitive). The age and sex-matched control group consisted of able-bodied individuals. These participants were recruited for convenience, from those who attended the institution.

Inclusion criteria were (1) written informed consent; (2) a confirmed clinical diagnosis of an exclusively motor impaired ataxia; (3) adults aged 18–65 years old; (4) normal visual acuity even with the use of corrective lenses; and (5) no acute or chronic health conditions that could compromise the ability to use a computer keyboard, visual acuity less than 20/40 on the Snellen Scale [23–25], and a score below 25 points on the Mini-Mental State Examination (MMSE) [26, 27].

Exclusion criteria included (1) neurological comorbidities, including impaired ability to understand the tasks, visual impairment that prevented task performance, dystonia, and epilepsy; (2) people who did not understand the proposed activities; (3) people who were unable to perform the tasks.

We estimated the sample size required in this study using a sample size calculation (software G*Power 3.1.5), and considering the main outcome measure (i.e., anticipation movement through error measures). This calculation was based on data from five patients (pilot study), considering a power of 0.80; α of 0.05, and effect size of 0.65 (Cohen d). The sample estimation indicated that 16 participants would be necessary in each group (i.e., 8 per sequence), and with an adjustment to allow for a withdrawal rate (20%), we included 33 participants.

### Sample characterization

The first stage of the protocol included the characterization of the sample through interviews. In this first visit, the participants answered a questionnaire that contained information about sociodemographic variables, technological profiles, and general health conditions. Next, the clinical and functional assessments were performed using the International Cooperative Ataxia Rating Scale (ICARS) [23, 24], the scale for the Assessment and Rating of Ataxia (SARA) [25–27], Snellen Scale for Visual Acuity [23–25], Mini-Mental State Examination (MMSE) [26, 27], Global Side Preference by IPLAG software [28], grip and pinch strength using a dynamometer [29–31], manual dexterity by the box and block [32, 33] and nine hole peg test [34]. This first visit lasted 1 hour and 30 minutes.

**Fig 1:**
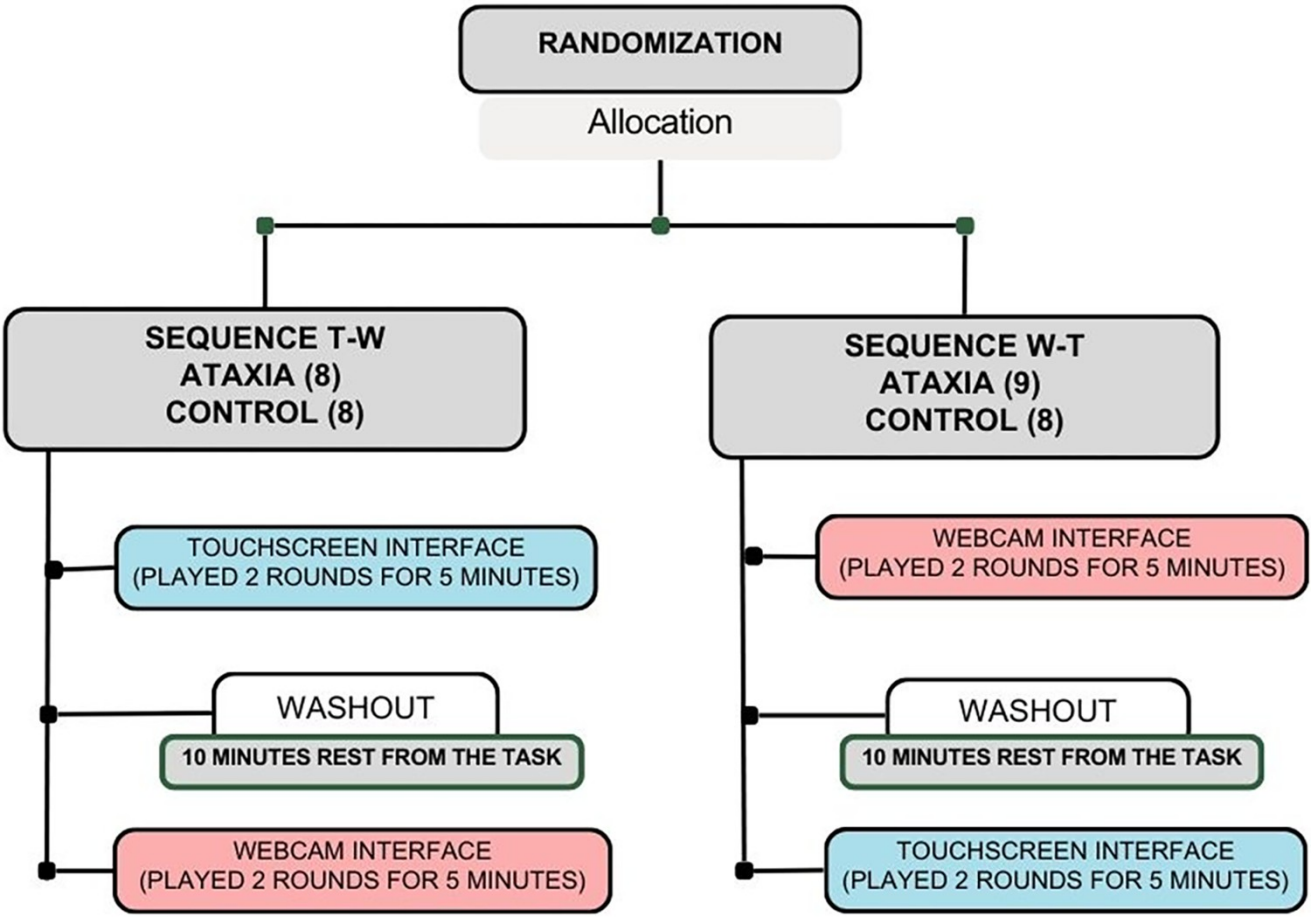
Virtual practice protocol.

After clinical evaluation of 36 participants, 33 met the inclusion criteria. For virtual practice and comparative analyses, the participants of the control group (16) were allocated to T-W (8) and W-T (8). In the group with Ataxia, 8 participants were allocated to T-W and 9 to W-T. On the second day and after receiving instructions, the participants performed the virtual games with webcam and touchscreen interfaces. The experiment lasted 1 hour and 20 minutes on average, and the participants were given a 10-minute rest interval between the virtual practices (Fig 1).

### Virtual reality (VR) tasks

The participants were comfortably positioned on a chair that was adjusted according to their size and needs. Before starting the task, the participants received instructions on how to play the VR game, and a demonstration was given by the examiner.

For virtual practices, we used *MoveHero Software (**https*:*//movehero*.*com*.*br/en/**)*, developed by the School of Arts, Sciences, and Humanities of the University of São Paulo and previously adopted in different studies [35–39]. In this online game, spheres of different colors appeared in a downward motion on the computer screen, synchronized with the rhythm of the music selected by the researcher. To avoid influencing the results, both the number of spheres and their speed were kept consistent throughout the entire protocol. These spheres move towards four targets labeled A, B, C, and D. Targets A and D are positioned laterally on the left and right sides, respectively, while B and C are centrally located, with B on the left and C on the right. The sequence of targets begins on the lateral left (A), followed by the central left (B), central right (C), and ends on the lateral right (D). To successfully perform the game, the participant is required to touch the spheres when they reach one of the four circles presented on the computer screen. The fixed circles are in parallel (at two height levels), two on the left and two on the right of the participant, and are denominated targets 1, 2, 3, and 4 (from left to right).

**Fig 2:**
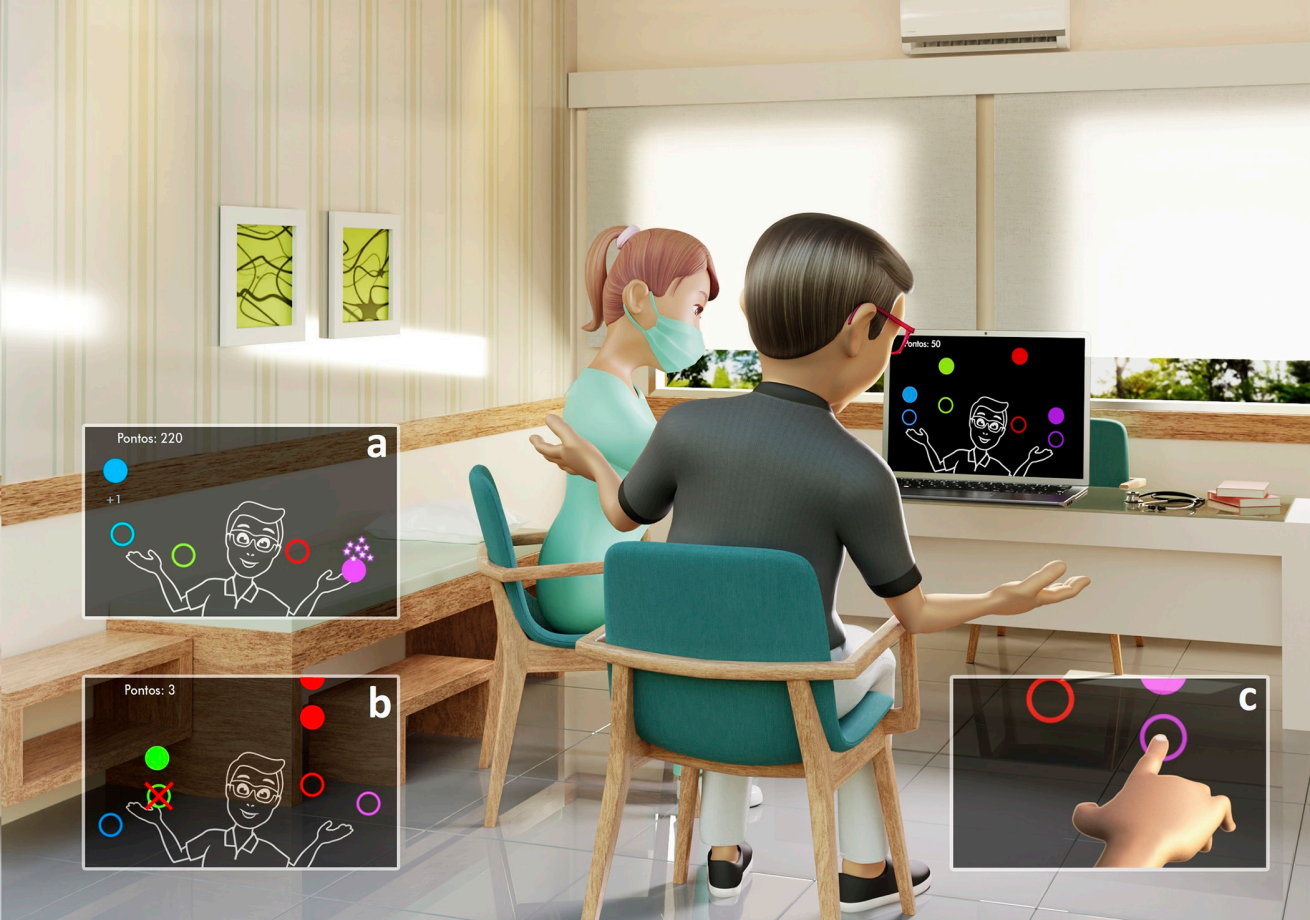
Representative design of the MoveHero software using the webcam to capture the body’s movement. (a) Demonstration of a hit performed by the participant (highlighting the sphere reached). (b) Demonstration of error performed by the participant (red X on the sphere). (c) Demonstration of the task using the touchscreen.

To perform the virtual game, the webcam was activated to capture the participant’s movements. The participant moves their upper limbs at a distance of 0.5–1 meter from the computer screen in order to catch the moving targets. When the participant hits the target, a visual signal alerts them to the number of hits. In addition, the total score is visible in the upper left corner of the screen, with 10 points for each hit. For the practice with physical contact, the touchscreen is active, which serves as tactile feedback. The score varies according to the rhythm of the chosen song, so that the maximum score possible is between 1040 and 1080, as illustrated in Fig 2.

### Statistical analysis

Descriptive statistics were initially used to describe data on socioeconomic profile and technological knowledge, in addition to specific tests for the characterization of the Ataxia Group. The frequency and/or means and standard deviation of the variables were reported. For the independent variables of the Ataxia and Control Groups, the Student’s t-test was used.

The dependent variable, obtained in the virtual task, was the error, defined as the difference between the time when the sphere reached the target (time of arrival) and the time when the participant hit the sphere (in milliseconds). From these times, we computed the Absolute Error (AE), which reflects the movement accuracy, it is calculated by taking the absolute value of each raw score, ignoring whether the response was early or late, high absolute error values ​​represent worse accuracy, and the Variable Error (VE), which can be defined as movement precision, it represents the timing error due to within-subject variability, and is calculated as the square root of the sum of the squared differences between each score and the individual’s constant mean, divided by the number of trials, high variable error values ​​represent worse precision. We also computed the number of hits (spheres hit on target) and anticipations (attempts made before the sphere hit the target) during the game [35, 40–42].

The dependent variables are presented as mean and standard deviation for the "MoveHero" game. MANOVA was employed with 2 (Group: Ataxia and Control) by 2 (Interface: Touchscreen—webcam), by 4 (Positions: A, B, C, and D), by 2 factors (Sequence: T-W = Touchscreen–Webcam; W-T = Webcam—Touchscreen), with repeated measurements in the last two factors. Post hoc comparisons were performed with the LSD (Least Significant Difference) test. The effect sizes from different comparisons are reported (ŋ_p_^2^) and interpreted as small (effect size >0.01), medium (effect size >0.06), or large (effect size >0.14) [43].

Additionally, to explore the factors influencing the results, we analyzed a pearson correlation between the change in motor scores (calculated as the difference between the second and first practice attempts for each target position) and the scores from the strength and coordination tests (Grip Strength, Pinch Strength, Box and Block Test, Nine Hole Peg Test), as well as the ataxia scores measured by both the SARA and ICARS scales. For all statistical analyses, we adopted an α = 0.05. The statistical package used was SPSS, version 26.0. All data from this research are publicly available [44].

## Results

### Sample characterization

In total, 16 individuals were included in the Control Group and 17 individuals with a confirmed diagnosis of Genetic Ataxia. The demographics of the group with Ataxia were as follows: 55% were female, with a mean age of 41.4 years; 55% received disability pensions; 33% attended higher education, and 44% had completed high school; 100% lived in their house; 66% reported needing a caregiver; and 88% used the public health network and did not have medical insurance.

Among genetic ataxic patients, 27% had Friedreich’s Ataxia, 44.4% Machado Joseph’s disease (SCA 3), 5.5% Friedreich-like Ataxia with selective vitamin E deficiency, 11.1% spinocerebellar ataxia type 1 (SCA1), and 16% presented rare unconfirmed forms. Regarding the performance on the specific scales for ataxia, the mean total score on SARA was 16.27 (±7.7) points and on ICARS was 19.22 (±9.7).

Regarding functional classification, all participants presented visual acuity considered normal with corrective lenses, and varying levels of dependence due to the progressive character of the conditions. Considering mobility, 27% were wheelchair-restricted, 50% walked with auxiliary devices, and 22% had independent but unstable gait. In addition, all participants presented enough manipulative ability to practice computational and virtual tasks.

Regarding the technological profile, 50% reported using at least three electronic devices in day-to-day life, with the cell phone as the main equipment used to search for information and communication. In addition, 50% had no habit of playing virtual games, and 38% preferred and downloaded games for mobile or tablet.

Considering the independent variables, a significant difference was found for the scores of the manual dexterity tests, with both values being higher in the Control Group. The data are shown in Table 1.

**Table 1:** Mean and standard deviation (SD) of the independent variables for control and ataxia groups.

	ATAXIA	CONTROL	P value
**AGE**	36.4 ± 12.2	41.4 ± 12.0	0.247
**GSP-IPLAG**	3.7 ± 0.6	3.8 ± 0.4	0.579
**GRIP S (R)**	29.2 ± 8.6	28.7 ± 9.7	0.889
**GRIP S (L)**	26.9 ± 8.7	26.2 ± 9.4	0.839
**PIN S (R)**	11.23 ± 3.2	9.8 ± 3.5	0.382
**PIN S (L)**	9.4 ± 2.6	8.5 ± 2.7	0.488
**BBT (R)**	66.8 ± 13.2	44.1 ± 18.3	< 0.001*
**BBT (L)**	62.62 ± 11.3	40.4 ± 15.8	< 0.001*
**9HP (R)**	19.80 ± 10.1	40.4 ± 19.4	0.001*
**9HP (L)**	22.4 ± 10.6	43.4 ± 17.4	< 0.001*

GSP–IPLAG: Global Side Preference; GRIP S: *Grip* strength, PIN S: Pinch strength; BBT: Box and *Block Test*; 9HP: Nine Hole Peg test; R: Right; L: Left; Bold: p < 0.05.

### Virtual practice

MANOVA revealed a main effect of Group [F_2, 26_ = 8,85; p = 0,001, ŋ_p_^2^ = 0,405; Wilks’ λ = 0.595]. The Ataxia group exhibited lower performance during all tasks when compared to matched controls. MANOVA also revealed a main effect of position [F_6, 22_ = 23.72; p < 0.001, ŋ_p_^2^ = 0.866; Wilks’ λ = 0.134]. Ataxia and control groups performed better in the central targets than peripheral targets.

MANOVA revealed a marginal interaction between Sequence * Group [F_2, 26_ = 3.30; p = 0.053, ŋ_p_^2^ = 0.203; Wilks’ λ = 0.797]. An interaction was also found between Sequence * Interface [F_2, 26_ = 70.69; p < 0.001, ŋ_p_^2^ = 0.845; Wilks’ λ = 0.155]. Finally, MANOVA revealed an interaction between Sequence * Interface * Position [F_6, 22_ = 6.80; p < 0.001, ŋ_p_^2^ = 0.650; Wilks’ λ = 0.350].

#### Absolute error (accuracy)

ANOVA revealed an interaction between Sequence * Group [F_1, 27_ = 6.83; p = 0.014, ŋ_p_^2^ = 0.202]. The post-hoc test showed that the Ataxia group exhibited higher AE in the T-W sequence (touchscreen–webcam) than in the W-T sequence (webcam—touchscreen) (p = 0.030), while no significant differences were found for the control group (p = 0.174).

An interaction was also found between Sequence * Interface [F_1, 27_ = 134.08; p < 0.001, ŋ_p_^2^ = 0.832]. The post-hoc test revealed that both groups presented a higher AE with the webcam interface compared to touchscreen during the T-W sequence [(Controls: *Touchscreen* M = 221 ms, *Webcam*: M = 207 ms; p = 0.001); (Ataxia: *Touchscreen* M = 315 ms, *Webcam*: M = 380 ms); p = 0.019)]. In addition, all participants presented a higher AE for the webcam interface than the touchscreen in the W-T sequence (touchscreen—webcam) (Controls: *Touchscreen* M = 140 ms, *Webcam* M = 303 ms; p < 0.001); [(Ataxia: *Touchscreen*: M = 241 ms, *Webcam* M = 519 ms; p < 0.001)].

**Fig 3:**
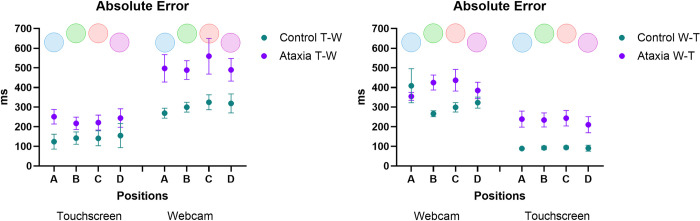
Mean and standard error of Absolute Error (AE, i.e., accuracy) in milliseconds of both groups (Ataxia and Control) and subgroups (T-W: touchscreen—Webcam and W-T: Webcam—touchscreen), and in the four targets position (A, B, C and D).

ANOVA revealed an interaction between Group * Sequence * Interface * Position, [F_3, 81_ = 3.78; p = 0.023, ŋ_p_^2^ = 0.123]. The *post-hoc* test showed that in the W-T sequence, the control group presented a lower AE throughout the sequence, in both interfaces and in all positions, compared to the Ataxia group (Fig 3).

#### Variable error (precision)

**Fig 4:**
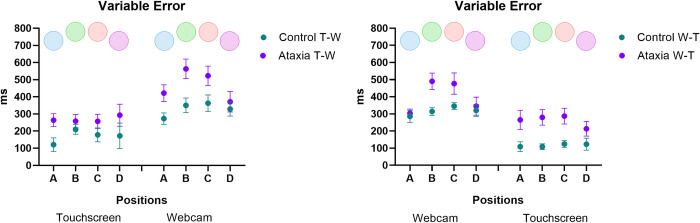
Mean and standard error of variable error (VE, i.e., precision) in milliseconds of both groups (Ataxia and Control) and subgroups (T-W: touchscreen—webcam and W-T: webcam- touchscreen), and in the four targets position (A, B, C and D).

Fig 4 presents the variable error (VE) data. A significant main effect was found for the Position of the targets [F_3, 81_ = 9.31; p < 0.001, ŋ_p_^2^ = 0.257]. The *post-hoc test* showed that in the central positions (B and C), participants exhibited a higher VE when compared with the lateral positions (A and D). Significant differences were found for: target position B (M = 323 ms) vs. A (M = 257 ms), and B vs. D (M = 276 ms), p values <0.016; target position C (M = 323 ms) vs. A (M = 257 ms), and C vs. D (M = 276 ms), p-values <0.006.

ANOVA also revealed an interaction between Sequence * Interface [F_1, 27_ = 111.57; p < 0.001, ŋ_p_^2^ = 0.805]. The *post-hoc* test showed that for the T-W sequence (touchscreen–webcam), the Ataxia group presented a higher VE in the webcam interface (M = 487 ms) compared to the touchscreen (M = 278 ms; p<0.001). A similar effect was found for the control group in the T-W sequence (webcam: M = 328 ms; touchscreen: M = 170 ms; p < 0.001). In the W-T sequence (webcam–touchscreen), the ataxia group also presented a higher VE in the webcam interface (M = 401 ms) compared to the touchscreen (M = 262 ms; p < 0.001). A similar effect was found for the control group in the W-T sequence (webcam: M = 316 ms; touchscreen: M = 116 ms; p < 0.001).

ANOVA showed interactions between Sequence * Interface * Position [F_3, 81_ = 6.79; p < 0.001, ŋ_p_^2^ = 0.201], and between Group * Sequence * Interface * Position [F_3 81_ = 4.89; p = 0.005, ŋ_p_^2^ = 0.153]. The *post-hoc* test revealed that Controls performed better during the T-W sequence in the touchscreen interface for target A than B (p = 0.034). Controls also performed better during the W-T sequence in the touchscreen interface for target A than C (p = 0.040). The *post-hoc* test showed that the Ataxia group presented better performance during the W-T sequence in the webcam interface for target A compared to B and C (p = 0.001 and p = 0.007, respectively) and for target D compared to B and C (p≤0.001). The Ataxia group also exhibited better performance during the W-T sequence in the webcam interface for target A compared to B and C (p <0.001) and for target D compared to B and C (p = 0.009 and p = 0.005, respectively).

#### Number of hits and anticipations during the virtual practice

MANOVA revealed a significant main effect of Group [F_2, 28_ = 12.63; p < 0.001, ŋ_p_^2^ = 0.474; Wilks’ λ = 0.526] and position [F_6, 24_ = 109.22; p < 0.001, ŋ_p_^2^ = 0.965; Wilks’ λ = 0.035]. A marginal effect was found for Sequence [F_2, 28_ = 2.94; p = 0.069, ŋ_p_^2^ = 0.174; Wilks’ λ = 0.826]. MANOVA also revealed an interaction between Sequence * Group [F_2, 28_ = 4.41; p = 0.022, ŋ_p_^2^ = 0.240; Wilks’ λ = 0.760], between Sequence * Interface [F_2, 28_ = 75.37; p < 0.001, ŋ_p_^2^ = 0.843; Wilks’ λ = 0.157], between Sequence * Interface * Position [F_6, 24_ = 3.40; p = 0.014, ŋ_p_^2^ = 0.460; Wilks’ λ = 0.540], and between Group * Sequence * Interface * Position [F_6, 24_ = 3.23; p = 0.018, ŋ_p_^2^ = 0.447; Wilks’ λ = 0.553].

#### Number of hits

ANOVA revealed a significant main effect for Position [F_3, 87_ = 226.28; p < 0.001, ŋ_p_^2^ = 0.886]. The *post-hoc* test showed that fewer hits were observed in the lateral positions (A and D). Significant differences for the Position of the targets A and D were found compared to other positions: position A (M = 14.0) and position B (M = 23, 1; p < 0.001), position A and C (M = 22.7; p < 0.001), position A and D (M = 149; p < 0.001), position D and B (p < 0.001), and position D and C (p < 0.001). There was also an interaction between Position * Group [F_3, 87_ = 3.14; p = 0.035, ŋ_p_^2^ = 0.098]. In all positions, the control group had more hits than the Ataxia Group (p < 0.001).

ANOVA revealed an interaction between Sequence * Interface [F_1, 29_ = 125.37; p < 0.001, ŋ_p_^2^ = 0.812]. The *post-hoc* test showed that in the T-W sequence (touchscreen–webcam), the Ataxia group decreased the number of correct hits in the webcam interface compared to the touchscreen (M = 11.7 and M = 18.2, respectively; p < 0.001). A similar effect was found for the control group in the T-W sequence (webcam: M = 19.0; touchscreen: M = 24.1; p < 0,001). In the W-T sequence (webcam–touchscreen), the ataxia group also presented fewer correct hits in the webcam interface compared to the touchscreen (M = 11.8 and M = 20.5, respectively; p < 0.001). A similar effect was found for the control group in the W-T sequence (webcam: M = 18.2; touchscreen: M = 25.6; p < 0,001).

ANOVA revealed an interaction for Sequence * Interface * Position [F_3, 87_ = 3.62; p = 0.026, ŋ_p_^2^ = 0.111] and group * Sequence * Interface * Position [F_3, 87_ = 3.48; p = 0.030, ŋ_p_^2^ = 0.107]. For the controls, in the T-W sequence and the W-T sequence in the touchscreen interface, fewer hits were observed for the target Position A compared to B and C (p-values <0.001) and for target D compared to B and C (p-values <0.001). A similar effect was found for the webcam interface in the T-W sequence and the W-T sequence. Fewer hits were found on target A compared to B, C (p-values <0.001), and D (p-values≤0.003), and for target D compared to B and C (p-values <0.001).

**Fig 5:**
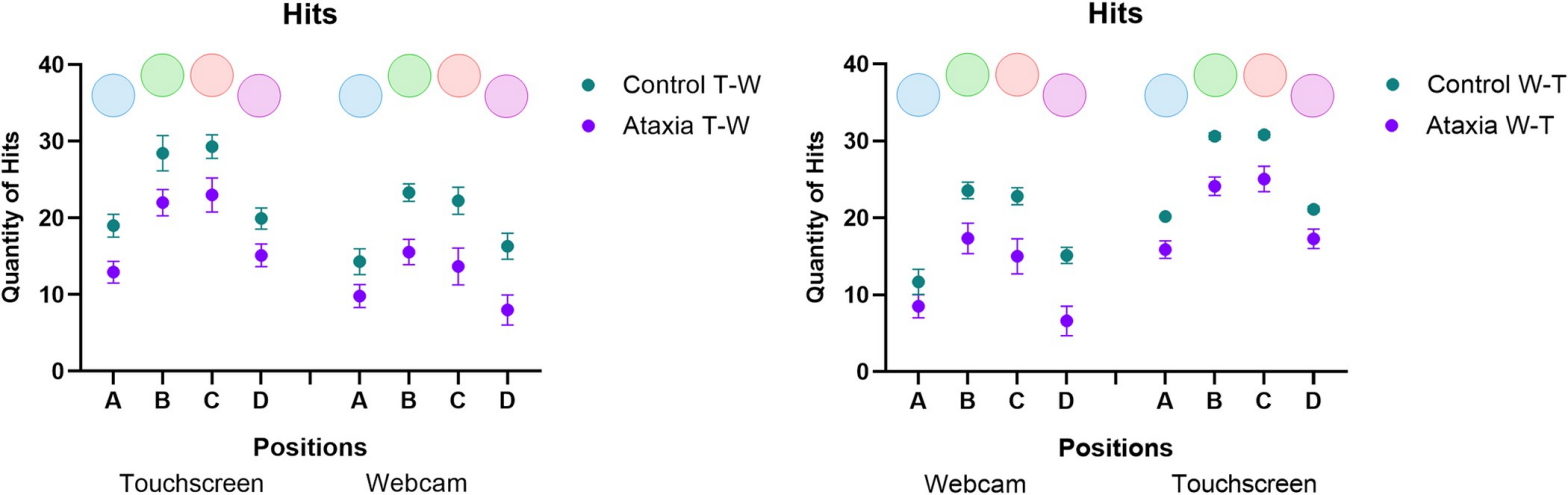
Mean and standard errors for the number of correct answers for both groups (Ataxia and Control) and subgroups (T-W: touchscreen—webcam and W-T: webcam—touchscreen), and in the four targets position (A, B, C and D).

For the Ataxia group, in the T-W sequence and the W-T sequence in the touchscreen interface, fewer hits were found for target A compared to B, C (p-values<0.001 and p-values<0.009, respectively), and D (p = 0.048). During both the T-W sequence and the W-T sequence, there were fewer hits on target D compared to B and C (p-values ≤ 0.001). A similar effect was found for the webcam interface in the T-W sequence and the W-T sequence. Fewer hits were found on target A compared to B and C (p-values <0.001) and for target D compared to B and C (p-values <0.001). In general, both groups presented fewer hits when the targets were presented at lateral (A and D) than central (B and C) positions for all experimental conditions (Fig 5).

#### Anticipations (attempts made before the spheres hit the target)

A significant effect was found for Position [F_3, 87_ = 9.83; p < 0.001, ŋ_p_^2^ = 0.253], The *post-hoc test* showed that in the lateral positions (A and D), there was less anticipation when compared with the central positions (B and C). Thus, a significant marginal difference was found between position A (M = 2.3) and position B (M = 3.0; p = 0.062), and a significant difference between positions A and C (M = 3.8; p = 0.004), as well as between positions D (M = 2.0) and B (p = 0.003), and positions D and C (p < 0.001).

**Fig 6:**
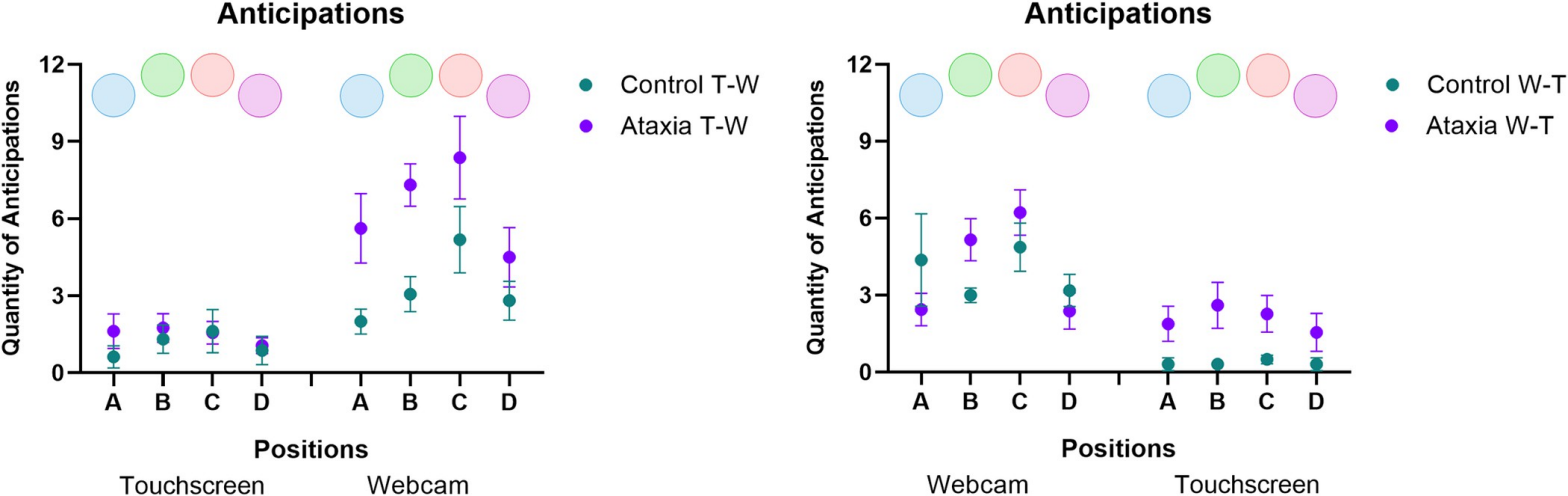
Mean and standard errors for number of anticipations for both groups (Ataxia and Control) and subgroups (T-W: touchscreen—webcam and W-T: webcam—touchscreen), and in the four targets position (A, B, C and D).

A significant interaction was observed between Sequence * Group [F_1, 29_ = 8.45; p = 0.007, ŋ_p_^2^ = 0.226]. The Ataxia group presented higher anticipation in the T-W sequence (touchscreen–webcam) than the W-T sequence (webcam—touchscreen), while no significant differences were found for the control group between the T-W sequence and in the W-T sequence (Fig 6).

ANOVA also revealed an interaction between Sequence * Interface [F_1, 29_ = 71.58; p < 0.001, ŋ_p_^2^ = 0.712]. The Ataxia Group in the T-W sequence (touchscreen—webcam) increased the amount of anticipation in the touchscreen (M = 3.4; p < 0.001) compared to the webcam (M = 1.5). A similar effect was found for the Control Group—the T-W sequence (touchscreen: M = 1.1; webcam: M = 3.2; p = 0.008). In turn, the Ataxia Group the W-T sequence (webcam—touchscreen) decreased the amount of anticipation in the webcam (M = 2.0; p = 0.010) compared to the touchscreen (M = 4.0). A similar effect was found for the Control Group the W-T sequence (webcam: M = 3.8; touchscreen: M = 0.3; p < 0.001).

### Correlation analysis

The control group T-W sequence shows significant correlations between absolute error and both BBT (negative) and 9HP (negative), as well as between the quantity of hits and 9HP (positive). This implies that higher absolute errors are associated with lower BBT and 9HP scores, while more hits are associated with higher 9HP scores. No correlation was observed in the control group for the W-T sequence, nor in either sequence for the ataxia group (Table 2).

**Table 2:** Correlation analysis.

**Pearson correlation**		**Control group—T-W Sequence**						**Control group—W-T Sequence**					
		GRIP S	PIN S	BBT	9HP	SARA	ICARS	GRIP S	PIN S	BBT	9HP	SARA	ICARS
**Absolute error**	r	.347	.099	**-.751**	**-.857** ^*^	-	-	.147	-.242	.346	-.527	-	-
	p	.501	.875	**.050**	**.014**	-	-	.752	.601	.401	.180	-	-
**Variable error**	r	.008	-.386	.504	-.682	-	-	.164	-.262	-.364	-.450	-	-
	p	.987	.521	.248	.092	-	-	.725	.571	.375	.263	-	-
**Hits**	r	-.495	-.195	-.563	**.817** ^*^	-	-	.305	.218	.086	.655	-	-
	p	.318	.754	.188	**.025**	-	-	.507	.639	.840	.078	-	-
**Anticipations**	r	-.220	.321	.208	-.260	-	-	.274	-.207	.450	-.250	-	-
	p	.675	.598	.655	.573	-	-	.552	.657	.264	.551	-	-
**Pearson correlation**		**Ataxia group—T-W Sequence**						**Ataxia group—W-T Sequence**					
		GRIP S	PIN S	BBT	9HP	SARA	ICARS	GRIP S	PIN S	BBT	9HP	SARA	ICARS
**Absolute error**	r	.226	.153	.194	-.116	-.708	.132	.477	.232	-.200	-.038	.206	-.291
	p	.626	.902	.678	.805	.075	.779	.232	.851	.635	.929	.625	.485
**Variable error**	r	.360	.968	.487	-.436	-.814*	.028	.442	-.972	-.452	.524	.302	.012
	p	.428	.162	.268	.328	.026	.952	.273	.150	.261	.182	.467	.977
**Hits**	r	-.358	-.264	-.274	.489	.641	.029	-.594	-.233	.111	-.225	-.351	.352
	p	.384	.736	.512	.218	.087	.946	.091	.851	.776	.561	.355	.353
**Anticipations**	r	.246	.179	.160	.089	-.555	.042	.161	-.421	-.390	.199	.334	-.224
	p	.556	.821	.705	.833	.153	.922	.678	.723	.300	.608	.380	.562

GRIP S: *Grip* strength, PIN S: Pinch strength; BBT: Box and *Block Test*; 9HP: Nine Hole Peg test; Bold: p < 0.05.

## Discussion

This study aimed to examine the effects of different interfaces (webcam and touchscreen) and the order of sequence practice (T-W or W-T) on the motor performance of individuals with genetic ataxia during a non-immersive virtual reality task. Our findings supported the initial hypotheses. The control group outperformed the ataxia group across the entire protocol. Also, physical interaction with the touchscreen interface leads to better performance for all participants compared to the webcam interface. Furthermore, the order of practice influenced motor performance, with practicing the non-contact task first benefiting subsequent contact-based task performance. We will discuss these findings further below.

### Ataxia and control group

Our result revealed that the Ataxia group presented worse performance, with more absolute (accuracy) and variable (precision) errors than controls. The Ataxia group also exhibited fewer hits and higher anticipation error in both the webcam and touchscreen interfaces. We can only speculate that ataxia individuals frequently present permanent neurological impairment, associated with significant impairment of intra-limb coordination and postural control functions, and these factors contribute to impaired motor performance, with slower and less accurate and precise movements [45]. This can be seen in the results of the BBT and the 9HP where the Ataxia group presented lower scores compared to the control group. In the correlation analysis there is a significant correlation between absolute error and the BBT and 9HP score only for the control group.

Individuals with ataxia present altered and decomposed movement patterns that generate slower responses, easily missing the intended targets, and requiring multiple corrections. Moreover, the consequences generated by dysmetria and tremor, characteristic of Ataxia, explain the cause of more dysfunctional motor control [45]. Dysmetria stems from deficits in the predictive computation of the internal forward model in the cerebellum. Errors in this fundamental mechanism result in undershoot (hypometria) and overshoot during voluntary motor actions [46].

This same behavior was observed in the study of Kakei and contributors (2019), [47] in which the authors verified that the increased muscle activities of the patients with cerebellar Ataxia were characterized by a marked decrease in speed adjustment and a compensatory position adjustment, resulting in a series of irregular movements, with low accuracy. In contrast, the muscle activities of the control group during the range of movement allowed the adjustment of both the speed and position of the target, with consequent efficient tracking of the movement [47].

Although these studies justified better performance for the control group, our study revealed an interesting result: the Ataxia group appears to have better accuracy than precision across both interfaces, since mean values suggest lower absolute error than variable error. This pattern indicates that individuals with Ataxia tend to reach the intended target but exhibit inconsistent distances between attempts. This variability may be attributed to cerebellar dysfunction, as the cerebellum plays a key role in calculating movement trajectories toward a moving target. Effective targeting requires the ability to predict both the current and future states of the body and the target, ensuring coordinated movement and hits [48].

Moreover, the physiological delay in sensory feedback, in milliseconds, predicted in healthy people, may be higher in individuals with Ataxia, which leads to a greater number of errors, more oscillatory motion, and more unstable movements. Archambault (2015) [49] claims that feedforward processes are needed to plan reaching movements, while feedback is used to track errors when performing the movement and for monitoring the result [48, 49].

Additionally, visuomotor control should also be considered, since sudden changes in targets require rapid corrections of movements, which are usually more decomposed and slower in individuals with ataxia. We can hypothesize that the worse performance from the Ataxia group considering visuomotor difficulties can be justified by: (1) the presence of nystagmus, which contributes to the delay in the variation of responses toward the intended target; (2) visuomotor functions of the cerebellum, including control of vestibular ocular reflexes and tracing movements that are compromised in individuals with Ataxia; (3) alteration in saccadic movements and smooth pursuit that are important for locating static or moving targets, promoting distance, type of movement, and speed of target dysfunction in Ataxia individuals [49–51].

### Interface and order of presentation

Considering the interface and order of presentation, we observed two interesting results: (1) overall, participants from both groups performed better with the touchscreen interface, and (2) the group that initially practiced using the webcam interface demonstrated improved performance during subsequent touchscreen practice (W-T sequence).

(1) The better performance with the touchscreen can be justified by the increase in tactile feedback, which probably favored the predictive calculation of the movements. In addition, the webcam task offers less sensorial support for the execution of movements, and we can hypothesize and highlight the importance of visual and proprioceptive feedback for cortical regions to ascertain the positions of the target and hands and quickly program the actions necessary for the execution of the reaching task [50]. Due to spinocerebellar degeneration, which impairs proprioceptive feedback [52], participants may struggle to integrate sensory information effectively, particularly when reaching to virtual targets that lie beyond the visual range and require greater reliance on proprioception, leading to reduced accuracy and performance.

It is probable the two tasks (with or without physical contact) depend on different information–movement couplings and that the physical contact also generates tactile information which can be used to adapt the movement to the environment, providing better performance. Thus, a task that involves a direct interaction with the environment, including physical contact (as in the touchscreen task) generates a richer pool of information for guiding movement than a more abstract task in a virtual environment (as in the webcam task) [53].

(2) The most important result from our study was the influence of the order of the sequence (T-W or W-T), where the Ataxia group that started practicing with the webcam interface presented better performance. It is important to emphasize that this difference occurred only in the group with ataxia (i.e., this difference can be verified by the positive interaction between sequence and group, which presented a lower absolute error when practicing the sequence with the webcam first).

Thus, we can only hypothesize that, in individuals with ataxia, practicing a more difficult task first, such as with the webcam, can improve performance in a touchscreen task performed subsequently. According to several studies that analyzed the influence of the task difficulty in the improvement in performance, when practicing a more difficult task, the motor and cognitive demands can act as a stimulus that optimizes the adaptation to the task demand and can positively influence a similar and easier task practiced afterwards [51, 53–55]. According to Rietschel and contributors [54], practicing a more difficult task provides a greater ‘neural effort,’ increasing neural activation and cortical networking when compared to an easy condition (the study used EEG spectral power and cortical networking between the premotor and sensory regions in healthy people).

On the other hand, performing with the touchscreen interface first impaired performance in the webcam interface. The hypothesis for this behavior is that when starting with an easier task, an internal model and predictability are created, that the subsequent task will require the same motor control standards to hit the target. Familiarization with the initial task underestimates the later task (webcam) and generates more errors and loss of accuracy and precision. It is important to emphasize that the influence of the order of practice was only observed in the Ataxia group.

We can justify these behavioral observations in goal-directed tracking tasks in genetic ataxia patients as being related to the impairment in continuous predictive controls and the greater oscillations and intermittent corrections based on the sequence of movements performed in the first task. We argue that impairment in the predictive computation for voluntary movements explains a range of characteristics accompanied by dysmetria. Within this framework, the cerebellum acquires and maintains an internal forward model, which predicts the current and future states of the body by integrating an estimate of the previous state and a given efference copy of motor commands. In addition, it is expected that the role of the cerebellum in motor control is grading muscle tone and tuning other cortical and subcortical centers so that muscle contractions are appropriately graded. Such a refining function gives the cerebellum a response time control action. Thus, it is expected that patients with GA present a disproportionate increase in temporal variability during a reaching task, as confirmed in our initial hypothesis.

### Target position

We found another interesting result regarding the target position. Individuals with Ataxia presented better results for the center targets when compared with the lateral targets. Although these results are the same as the control group, this feature provides us with a more detailed analysis and the importance of the target task position in a rehabilitation setting to characterize difficulty.

The task used is a double-step virtual activity, where targets appear in a downward motion and are quickly replaced by others in unpredictable (left or right) directions. This dynamism requires predictive calculation, position judgments, monitoring, and online corrections for the target range [55, 56] and these task characteristics could be responsible for the difficulty with the lateral targets.

We can only speculate that the (1) lower saccadic movement and (2) smaller range of body movement necessary for closer targets are responsible for the differences presented in the target position.

(1) Lower saccadic movement: Butcher et al. (2017) [51] state that individuals with cerebellar injury present sensory-motor adaptation deficits and that error-based learning and the ability to develop and/or maintain a solution in response to a visuomotor disturbance to modify the internal model are impaired. Moreover, Junyu Lin (2021) [57] reported that abnormal eye movements are common in Hereditary Ataxias, including impaired smooth pursuit, increased square-wave jerks (SWJ), gaze-evoked nystagmus (GEN), slowing of saccades, saccadic hypo/hypermetria, and supranuclear gaze palsy. Therefore, central targets are closer to the participants and can be better perceived with lower saccadic movements of the eyes, facilitating motor control adjustments to hit the correct target.

Considering studies with Ataxia participants and eye movements, some authors reported that changing the position of the target occurs during a saccadic movement, that may not be immediately identified, which increases response time (latency) and decreases accuracy [55, 58]. Brenne and Smeets (2009) [56] complement that the greater interference in latency is related to the judgment of the new position, the orientation of the target, and the predictability of eye movements. Oostwoud Wijdenes et al. (2014) [55] show that eye movements are adjusted according to the position and moments of presentation of two targets in different locations. Thus, during the execution of distant targets (with more eye range of movement and difficulty predicting the target), the participant increased the saccadic movement, which could be responsible for lower accuracy and precision in the lateral targets.

(2) Smaller range of body movement: Another influence that provides better performance in closer targets could be a smaller range of upper limb movements to reach the target. Our results agree with the Silva et al. (2022) [59] study that used the same task with individuals with motor disabilities and reported that closer targets lead to better performance. It is probable that the dysmetria, tremor, and deteriorating motor coordination that characterize Ataxia could be responsible for the worse accuracy and precision of movements toward more distant objects, which could explain the difficulty with the lateral targets.

We have to consider that the movement deviation becomes more significant for actions performed in distant and unpredictable tasks. In this case, the group ataxia has more difficulty in determining a compensatory strategy during a distance movement, which could need more velocity of motor action. Some authors concluded that the ataxic path was the natural consequence of inappropriate programming of muscle activity and the result of proprioceptive feedback, incorrectly modifying the central commands. We agree with these authors who affirm that Ataxia individuals present malfunction in central programming and peripheral feedback, which underlie the irregular movements observed in dysmetria, and that these difficulties could affect distant targets [60–63].

### Limitation

Although we found interesting results in the current study, some limitations should be presented: (1) the most important limitation is the group characterization, with a small number of participants, the inclusion of both male and female individuals in the study, as well as individuals in different progression phases (i.e., the progression phase was identified through the lower limb difficulties, and we compensated this difficulty by using an upper limb task). Although we know that a bigger group with more homogeneity is important, GA is considered a rare genetic disease, and difficulties finding participants and transporting these individuals to a research center justify the sample in this study; (2) in this study we used some factors, such as delayed sensory feedback, visuomotor dysfunction, reduced range of motion, impaired proprioceptive feedback, and diminished saccadic movement, to explain the performance of the Ataxia Group. While these difficulties are characteristic of genetic ataxia, it can be difficult to have a clear individualized influence of those factors. Therefore, further studies could investigate the specifics of each factor and its impact on performance; (3) Although oculomotor abnormalities can contribute to visual disturbances, poor coordination, and balance problems, we did not analyze the influence of nystagmus, saccades, and ocular dysmetria on participant performance. These factors are important and could yield valuable insights, which should be explored in future studies; (4) In this study, we used a constant velocity for the objects throughout the entire protocol to ensure consistency in our findings. However, different object speeds could affect task predictability, potentially influencing accuracy and performance. Therefore, future studies should explore varying velocities to better understand their impact on performance and further validate the influence of this factor. (5) Lastly, we suggest that future studies, with a longer time of practice (longitudinal protocol) including different virtual reality tasks, should be performed in the future.

## Conclusion

This study concluded that the group of participants with Ataxia presented lower performance than the control group, regardless of the interface (webcam or touchscreen). Moreover, the feedback provided by the touchscreen interface may have facilitated the target being reached, so that it was easier to practice the task with the touchscreen than with the webcam interface, suggesting that this is less taxing for individuals with ataxia. Nevertheless, the most interesting result was that practicing with the webcam interface offers features that make the task more complex than touchscreen, with lower performance. However, this lower performance in the webcam facilitated performance in a subsequent touchscreen task in individuals with genetic ataxia. We propose that using a virtual webcam may have greater translation to other tasks and be worthy of further investigation for physical tasks.

## Supporting information

S1 Checklist Inclusivity in global research. Ethical considerations, permits, authorship and Human subject’s research.
(DOCX)
